# Drug resistance genes: *pvcrt*-*o* and *pvmdr*-*1* polymorphism in patients from malaria endemic South Western Coastal Region of India

**DOI:** 10.1186/s12936-018-2188-6

**Published:** 2018-01-19

**Authors:** Shiny Joy, Benudhar Mukhi, Susanta K. Ghosh, Rajeshwara N. Achur, D. Channe Gowda, Namita Surolia

**Affiliations:** 1 Molecular Biology and Genetics Unit, Jawaharlal Nehru Centre For Advanced Scientific Research, Jakkur, Bangalore, India; 2 Department of Biological Control, National Institute of Malaria Research, Poojanahalli, Bangalore, India; 3 Department of Biochemistry, Kuvempu University, Shivamogga District, Shankaraghatta, Karnataka India; 4 Department of Biochemistry and Molecular Biology, The Pennsylvania State University College of Medicine, 500 University Drive, Hershey, PA 17033 USA

**Keywords:** *Plasmodium vivax*, Chloroquine resistance markers, Copy number variation, Mangaluru city area, India

## Abstract

**Background:**

Malaria is highly prevalent in many parts of India and is mostly caused by the parasite species *Plasmodium vivax* followed by *Plasmodium falciparum*. Chloroquine (CQ) is the first-line treatment for blood stage *P. vivax* parasites, but cases of drug resistance to CQ have been reported from India. One of the surveillance strategies which is used to monitor CQ drug resistance, is the analysis of single nucleotide polymorphisms (SNPs) of the associated gene markers. Susceptibility to CQ can also be determined by copy number assessment of multidrug resistant gene (*mdr*-*1*). The current study has examined the prevalence of SNPs in *P. vivax* orthologs of *P. falciparum* chloroquine resistant and multi-drug resistant genes (*pvcrt*-*o* and *pvmdr*-*1*, respectively) and *pvmdr*-*1* copy number variations in isolates from the highly endemic Mangaluru city near the South Western Coastal region of India.

**Methods:**

A total of 140 blood samples were collected from *P. vivax* infected patients attending Wenlock Hospital Mangaluru during July 2014 to January 2016. Out of these 140 samples, sequencing was carried out for 54 (38.5%) and 85 (60.7%) isolates for *pvcrt*-*o* and *pvmdr*-*1*, respectively. Single nucleotide polymorphisms (SNPs) in the *pvcrt*-*o* and *pvmdr*-*1* genes were analysed by direct sequencing method, while copy number variations of 60 isolates (42. 8%) were determined by real time PCR.

**Results:**

Out of 54 clinical isolates analysed for *pvcrt*-*o*, three (5.6%) showed K10 insertion and the rest had wild type sequence. This is the first report to show K10 insertion in *P. vivax* isolates from India. Further, out of 85 clinical isolates of *P. vi*vax analysed for mutations in *pvmdr*-*1* gene, only one isolate had wild type sequence (~ 1%) while the remaining (99%) carried mutant alleles. Seven non-synonymous mutations with two novel mutations (I946V and Y1028C) were observed. Of all the observed mutations in *pvmdr*-*1* gene, T958M was most highly prevalent (present in 90% of samples) followed by F1076L (76%), and Y976F (7%). Amplification of *pvmdr*-*1* gene was observed in 31.6% of the isolates, out of 60 amplified.

**Conclusion:**

The observed variations both in *pvmdr*-*1* and *pvcrt*-*o* genes indicate a trend towards parasite acquiring CQ resistance in this endemic area.

## Background

Malaria caused by *Plasmodium vivax* is a significant public health issue and is more prevalent than *Plasmodium falciparum* outside Africa, where *P. falciparum* is the predominant species [1]. Chloroquine and primaquine remains the first-line treatment for vivax malaria. Resistance to anti-malarial drugs is a major hurdle for malaria control strategies. In *P. falciparum*, decreased susceptibility to all the currently used anti-malarial drugs (amodiaquine, chloroquine, mefloquine, quinine, sulfadoxine-pyrimethamine) and more recently, resistance to artemisinin derivatives [2] has been observed and is a major concern. Although *P. falciparum* resistance to chloroquine (CQ) was reported in the 1950s, the first case of *P. vivax* resistance to CQ was reported only in 1989 from Papua New Guinea [3]. The mechanisms of *P. vivax* resistance to anti-malarials, have not been extensively studied due to the non-availability of continuous in vitro culture system. A majority of the reported studies have focused on analysing SNPs in resistant gene markers [4].

The *P. falciparum* orthologs for CQ-resistant genes, *pvcrt*-*o* and *pvmdr*-*1*, of *P. vivax* have been the main focus in studying the CQ resistance [5, 6]. A number of mutations in both genes have been reported. Among these, K10 insertion in *pvcrt*-*o* and several specific SNPs in *pvmdr*-*1* have been identified as possible molecular markers of CQ resistance in *P. vivax* [6, 7]. The Y976F and F1076L, which are non-synonymous amino acid mutations of the *Pvmdr*-1, have been reported to correlate with CQ resistance [8–10]. However, in some studies, no significant associations have been found between these mutations and clinical response of *vivax* malaria to chloroquine [5, 11, 12]. Further, as in *P. falciparum*, copy number variations in *pvmdr1* has been suggested to be associated with anti-malarial resistance [13, 14]. The *Pvmdr*-*1* gene amplification has been shown to correlate with susceptibility of *P. vivax* to various anti-malarial drugs [10]. This amplification leads to decreased susceptibility to mefloquine and increased susceptibility to CQ [15].

India is one of the countries where 80% of the total malaria cases occur worldwide due to *P. vivax* [1]. There have been several studies reporting CQ resistance in *P. vivax* resulting in fatal malaria in India [16–19]. Mangaluru is a coastal city in Southern Karnataka state of India that has malarial resurgence since 1990. Mangaluru city and its surrounding region are highly malaria endemic. Despite high endemicity and huge health burden, no systematic epidemiological studies with respect to disease severity, clinical manifestations and drug resistance has been conducted in this region. The major malaria parasite in this region is *P. vivax* (~ 80%). This study is the first attempt to analyse vivax gene markers implicated in isolates obtained from this region.

## Methods

### Sample collection and ethics

The study was conducted at Wenlock District Hospital, Mangaluru. Written consent from participants were taken and blood samples were collected during the period June 2014 to December 2015. Thick and thin smears obtained by finger prick were prepared on glass slides, stained with Giemsa, and examined under light microscopy. Blood samples were collected on Whatman No. 3MM filter paper, allowed to air-dry, placed individually in plastic bags and stored at − 80 °C until processed. A total of 140 *P. vivax* positive patients (as confirmed by microscopy, RDT and 18srRNA amplification by PCR) were included in this study. The study design was in accordance with the ethical guidelines of Indian Council of Medical Research (ICMR) and the National Institutes of Health, USA. The study protocols were approved by the Institutional review board of Kuvempu University, Shivamogga, Karnataka, India, and Pennsylvania State University College of Medicine, Hershey, PA, USA.

### DNA extraction and species confirmation

Genomic DNA was extracted from filter paper spots using Chelex-100 reagents (Chelex^®^-100, Himedia, USA) as described [20]. Briefly, each filter paper punch was incubated with 0.5% saponin in phosphate buffered saline at 4 °C overnight. The punches were washed with 1× PBS for 30 min, transferred to new tubes containing 5% Chelex-100 and vortexed for 30 s; followed by heating at 99 °C for 15 min and centrifuged at 14 K for 2 min. Supernatant was collected and stored at − 20 °C until used.

*Plasmodium vivax* infection identified by microscopy was confirmed by PCR analysis of 18 s ribosomal RNA of parasites in all samples. One genus-specific *(Plasmodium)* and two species-specific (falciparum and vivax) sets of primers were used for PCR analysis, as described previously [21].

### Amplification of chloroquine resistance associated genes

The *pvcrt*-*o* and *pvmdr*-*1* genes were amplified using primers and reaction conditions as described [22, 23]. Amplification of target gene fragments were performed by PCR using 30 ng template DNA and Phusion^®^ High-Fidelity DNA Polymerase. The PCR cycle conditions were as follows: initial denaturation at 98 °C for 30 s, 30 cycles of denaturation at 98 °C each for 10 s, annealing at primer-dependent temperature for 30 s, and extension at 72 °C for 1.15 min followed by final extension of 72 °C for 5 min. Optimal annealing temperature was 61 °C for both the genes.

### Sequence analysis

The PCR amplified gene products were extracted from gels using Gel Extraction kit (Sigma-Aldrich, St Louis, MO, USA) and the extracted DNA was quantified by nanodrop. Sequencing of genes from each isolate was performed on an ABI Prism 377 DNA Sequencer equipped with semi adaptive version 3.0. Nucleotide sequences were analysed using blast and Bio Edit Sequence Alignment Editor and compared with reference sequences of Gen-Bank accession numbers, AF314649 and AY571984 for *pvcrt*-*o* and *pvmdr*-*1*, respectively. Amplification and sequencing were repeated to confirm that the observed SNP variants were not due to PCR or sequencing errors.

### Determination of *pvmdr*-*1* gene copy numbers

*Pvmdr*-*1* copy number was determined by SYBR green based quantitative PCR using the primers as described [15]. Amplified products of *pvmdr*-*1* and *pvaldolase* (as internal control) were cloned (InsTAclone PCR Cloning Kit (Thermo Scientific Company, USA) into the vector pTZ57R/T and used as calibrators for the assay. For both the genes, 20 µL reaction contained of 10 µL of SYBR green mix (Bio-Rad Laboratories, USA), 0.125 µM of each primer and 1–10 ng genomic DNA. Amplification included a template denaturation step for 4 min at 95 °C, followed by 38 cycles of 95 °C for 30 s and 60 °C for 30 s and 72 °C for 30 s with fluorescence acquisition at the end of each extension step. Amplification was immediately followed by melting at 65–95 °C with stepwise temperature increase of 0.5 °C with fluorescence acquisition at each temperature transition. The assays were repeated. A copy number of < 1.6 was considered a single copy, and a copy number of ≥ 1.6 was considered multiple copies.

## Results

### Analysis of *pvcrt*-*o* gene polymorphisms

The *pvcrt*-*o* gene from 54 isolates of *P. vivax* from Mangaluru were sequenced. Of the 54 isolates analysed, wild type sequence was observed in 51 isolates (94%) and 3 isolates (5.5%) showed insertion of three bases, AAG, leading to K10 insertion in the first exon when compared with reference *pvcrt*-*o* sequence.

### Analysis of *pvmdr* -*1* gene polymorphisms and copy number assessment

The *pvmdr*-*1* gene amplified from 85 isolates was sequenced. Seven non-synonymous mutations (I946V, T958M, Y976F, F979S, M980V, Y1028C and F1076L) were observed. Among these, I946V and Y1028C mutations were observed for the first time from this region. Prevalence of the Y976F mutation was found to be 7.1% while F1076L mutation was 54.5%. The most prevalent and dominant (90.6%, n = 77) mutation was T958M (Table 1). The F979S and M980V mutations were observed in one sample (1.2% prevalence) alone. *Pvmdr*-*1* from one isolate had 100% homology with wild type gene sequence.

**Table 1:** Non synonymous mutations observed among isolates

No	Non synonymous mutations	Isolates N (%)
1	T958M	77 (90.6)
2	F979S	1 (1.2)
3	M980V	1 (1.2)
4	F1076L	65 (76.5)
5	I946V	1 (1.2)
6	Y976F	6 (7.1)
7	Y1028C	1 (1.2)

Ten different haplotypes of *pvmdr*-*1* were observed including one wild type. Single mutants either 958_M_ or 1076_L_, double mutants 958_M_ 976_F_, 958_M_ 1076_L_ and 976_F_ 1076_L_; triple mutants 958_M_ 976_F_1076_L_, 958_M_ 1028_C_ 1076_L_ and 946_V_ 958_M_ 1076_L_; and quadruple mutants 958_M_ 979_S_ 980_V_ 1076_L_ in different frequencies (Table 2).

**Table 2:** Different haplotypes having mutations in *Pvmdr*-*1* gene

Haplotype	Isolates number (%)
958_M_	16 (18.8)
1076_L_	6 (7)
958_M_976_F_	3 (3.5)
958_M_ 1076_L_	53 (62.4)
976_F_ 1076_L_	1 (1.2)
958_M_ 976_F_ 1076_L_	2 (2.4)
958_M_ 1028_C_ 1076_L_	1 (1.2)
946_V_ 958_M_ 1076_L_	1 (1.2)
958_M_ 979_S_ 980_V_ 1076_L_	1 (1.2)

### *Pvmdr*-*1* copy number assessment

The *pvmdr*-*1* copy number was analysed for 60 isolates. Of these, a majority of isolates (68.3%, n = 41) had single gene copy while the remaining (31.6%, n = 19) had more than one, i.e.; two, three or four copies.

## Discussion

This study has identified CQ resistance-associated genotypes of *P. vivax* in the highly malaria endemic area of Mangaluru city and its surrounding regions. Importantly, this is the single study so far to analyse the drug resistance associated SNPs from this region. The identified genotypes include those having SNPs in *pvcrt*-*o* and *pvmdr*-*1*, and also those with copy number variations in *pvmdr*-*1* gene. In case of *pvcrt*-*o*, 5.6% of isolates carried an insertion of three bases, namely AAG, in the first exon. This is the first study from India reporting K10 insertion in *pvcrt*-*o*. Earlier studies from Indonesia and Thailand reported a significant increase in CQ IC_50_ that correlated with K10 insertion and Y976F mutation in *pvcrt*-*o* and *pvmdr*-*1* respectively [24]. In our study, Y976F mutant was also observed among the isolates analysed with the prevalence rate of 7.1%. The previous clinical studies from India [8, 25] reported susceptibility towards CQ and the mutant Y976F was not observed in these two studies. The major haplotype in our study was found to be 958_M_ 1076_L_ similar to the studies reported earlier [25, 26]. This suggest that *P. vivax* isolates from Mangaluru are susceptible to CQ, but the occurrence of both Y976F mutants and K10 insertion though at a low rate may be an indication that chloroquine resistance in this geographical area will surface in near future.

Two novel mutations, I946V and Y1028C are observed in *pvmdr*-*1* at the rate of 1.2% (n = 1). While Y1028C is present in the extracellular loop and the other mutation, I946V is on the transmembrane domain of the *pvmdr*-*1* gene where the other reported T958M mutation is present. T958M mutation is observed in majority of the samples (90.6%) analysed in this study. Since T958M mutation is present in isolates from countries having low to high levels of CQ resistance, T958M appears to be an allelic variant of the wild type and is most likely not associated with CQ resistance [9, 12, 27]. The other two rare mutations, F979S, M980V, observed in this study were also found in isolates from Nepal [28]. This similarity in parasite population is may be due to the geographical similarity between India and Nepal. Overall the role of these mutations cannot be determined until they correlate with clinical studies.

Among 60 isolates for which copy number of *pvmdr*-*1* gene was analysed, more than half (68.3%) of the isolates have single copy gene and the remaining 31.6% carried multiple copies. Studies have reported that multiple copies inversely correlate with CQ resistance. So, increased percentage of isolates having single copy suggest decreased susceptibility to CQ. However, in vitro together with clinical phenotypic studies are required to confirm the drug susceptibility in this region. Further, since phenotypic studies have not been well established for *P. vivax*, results presented here on *pvcrt*-*o* and *pvmdr*-*1* markers should serve as the base for future studies, monitoring drug resistance in this region. Also, though K-10 insertion is found less frequent, this calls for attention to conduct regular monitoring for the drug resistance in this region for decisions on drug policy.

## Conclusion

Results of the current study show that, the *pvcrt*-*o* and *pvmdr*-*1* gene variants implicated in *P. vivax* CQ resistance are less frequent in Mangaluru. This frequency is an indicator of low *P. vivax* drug resistance, though reflecting a beginning of the trend. Hence, continuous monitoring of drug resistant markers and therapeutic efficacy studies would be desirable for proper management and administration of anti-malarial drugs.

## Abbreviations

API: annual parasite index
CQR: chloroquine resistance
*Pvmdr*-*1*: *Plasmodium vivax* multidrug resistant 1 gene
*Pvcrt*-*o*: *Plasmodium vivax* chloroquine resistance transporter
WHO: World Health Organization
SNP: single nucleotide polymorphisms
PBS: phosphate buffered saline
